# Response: “Commentary: Peripartum Cardiomyopathy in Intensive Care Unit: An Update”

**DOI:** 10.3389/fmed.2016.00018

**Published:** 2016-05-03

**Authors:** Vesna Dinic, Danica Markovic, Nenad Savic, Marija Kutlesic, Radmilo J. Janković

**Affiliations:** ^1^Department for Anesthesiology and Reanimatology, Clinical Center Nis, Nis, Serbia; ^2^School of Medicine, University of Nis, Nis, Serbia

**Keywords:** peripartum cardiomyopathy, levosimendan, cardiomyopathy, pregnancy, echocardiography

We are very glad that our work got the attention of Dr. Biteker, and we wish to thank him for mentioning PPCM definition by ESC.

According to workshop recommendations of National Heart, Lung, and Blood Institute and Office of Rare Diseases (National Institutes of Health) “peripartum cardiomyopathy is defined on the basis of 4 criteria, adopted from work by Demakis et al. (1, 2) which include the development of the disease in the last month of pregnancy or within 5 months of delivery; absence of an identifiable cause of heart failure; absence of recognizable heart disease prior to the last month of pregnancy and LV systolic dysfunction demonstrated by classical echocardiographic criteria” (3). Time period from 1 month before delivery and 5 months after delivery was emphasized as an exclusion factor for other, previously undiagnosed or preexisting types of cardiomyopathies, which can be unmasked by hemodynamic changes during pregnancy. U.S. National Library of Medicine still supports this definition of PPCM (4). Unfortunately, at this moment, there is no consensus about the definition of PPCM in scientific community, and we think that both definitions of PPCM are equally acceptable.

Considering echocardiography, we think that echocardiographic cutoff should not always lead to underdiagnosis, especially not in cases of latent forms of PPCM without clinical symptoms.

Currently, there is no consensus on the appropriate duration of medical therapy in PPCM. No recommendations can be made because only limited prospective long-term data are available.

We appreciate data provided by Biteker et al.

Regarding levosimendan, there are case reports (5, 6) about its successful use in PPCM. The study by Biteker et al. definitely provides additional data on the value of levosimendan in the treatment of PPCM. Contrary to the case reports that we cited (5, 6) this study did not show any benefit from levosimendan. This study was though labeled as “small sample size, single center experience, open label design” by Desplantie et al. (7). We believe that one study cannot prove or disapprove the value of levosimendan in PPCM and that more research is necessary.

## Author Contributions

All aforementioned authors contributed significantly to the final design of manuscript.

## Conflict of Interest Statement

The authors declare that the research was conducted in the absence of any commercial or financial relationships that could be construed as a potential conflict of interest.
